# RNA-seq profiling reveals PBMC RNA as a potential biomarker for hepatocellular carcinoma

**DOI:** 10.1038/s41598-021-96952-x

**Published:** 2021-09-07

**Authors:** Zhiyi Han, Wenxing Feng, Rui Hu, Qinyu Ge, Wenfeng Ma, Wei Zhang, Shaomin Xu, Bolin Zhan, Lai Zhang, Xinfeng Sun, Xiaozhou Zhou

**Affiliations:** 1 Department of Liver Disease, Shenzhen Traditional Chinese Medicine Hospital, Futian District, Shenzhen, 518033 Guangdong Province China; 2 Department of Liver Disease, The Fourth Clinical Medical College of Guangzhou University of Chinese Medicine, Shenzhen, 518033 China; 3 State Key Laboratory of Bioelectronics, School of Biological Science and Medical Engineering, Southeast University, Nanjing, 210096 China

**Keywords:** Cancer genomics, Biological techniques, Transcriptomics

## Abstract

Hepatocellular carcinoma (HCC) is one of the most common malignant tumors and has extremely high morbidity and mortality. Although many existing studies have focused on the identification of biomarkers, little information has been uncovered regarding the PBMC RNA profile of HCC. We attempted to create a profile throughout using expression of peripheral blood mononuclear cell (PBMC) RNA using RNA-seq technology and compared the transcriptome between HCC patients and healthy controls. Seventeen patients and 17 matched healthy controls were included in this study, and PBMC RNA was sequenced from all samples. Sequencing data were analyzed using bioinformatics tools, and quantitative reverse transcription PCR (qRT-PCR) was used for selected validation of DEGs. A total of 1,578 dysregulated genes were found in the PBMC samples, including 1,334 upregulated genes and 244 downregulated genes. GO enrichment and KEGG studies revealed that HCC is closely linked to differentially expressed genes (DEGs) implicated in the immune response. Expression of 6 selected genes (SELENBP1, SLC4A1, SLC26A8, HSPA8P4, CALM1, and RPL7p24) was confirmed by qRT-PCR, and higher sensitivity and specificity were obtained by ROC analysis of the 6 genes. CALM1 was found to gradually decrease as tumors enlarged. Nearly the opposite expression modes were obtained when compared to tumor sequencing data. Immune cell populations exhibited significant differences between HCC and controls. These findings suggest a potential biomarker for the diagnosis of HCC. This study provides new perspectives for liver cancer development and possible future successful clinical diagnosis.

## Introduction

One of the major causes of cancer mortality in the world is HCC^1^. There have been many new breakthroughs in diagnosis and treatment, such as radiofrequency ablation (RFA) and microwave ablation (MWA), with corresponding efficacy, reproducibility, low complication rates, and availability^2^. Specifically, immunotherapy has conveyed remarkable clinical responses in cancer patients^3^. Development of cell therapies, antitumor vaccines, and new biotechnological drugs has shown promising results in preclinical studies^4,5^. Notably, once cancer cells metastasize, forecasts are unsatisfactory, and the majority of cancers cannot be targeted for treatment at this point. This is primarily due to factors such as delay in or absence of diagnosis^6–8^. Candidates for liver cancer are commonly patients with underlying liver diseases, such as hepatitis B virus (HBV) infection and cirrhosis^9^. More than half of sick HCC persons are diagnosed with advanced disease, restricting treatment options. Imaging diagnoses, such as positron emissions tomography (PET), for example, are very specific tools for diagnosing HCC, but they lack typical small to micrometastasis imaging features. Alpha-fetoprotein (AFP) and alkaline phosphatase (ALP or AKP) are commonly examined at the moment, but these biomarkers are unsuitable in clinical practice for the early diagnosis of HCC. AFP (threshold of 20 μg/mL) is reported to have a low sensitivity of 40–60% and a specificity of 80–90%^10–12^. False negatives (e.g., small HCCs with ordinary AFP standards), false positives (e.g., liver injury and certain gastrointestinal tumors) and low sensitivity may decrease the likelihood of early diagnosis, which could lead to worse clinical outcomes. Deterioration also emphasizes that HCC detection techniques need to be more specific.

**Figure 1:**
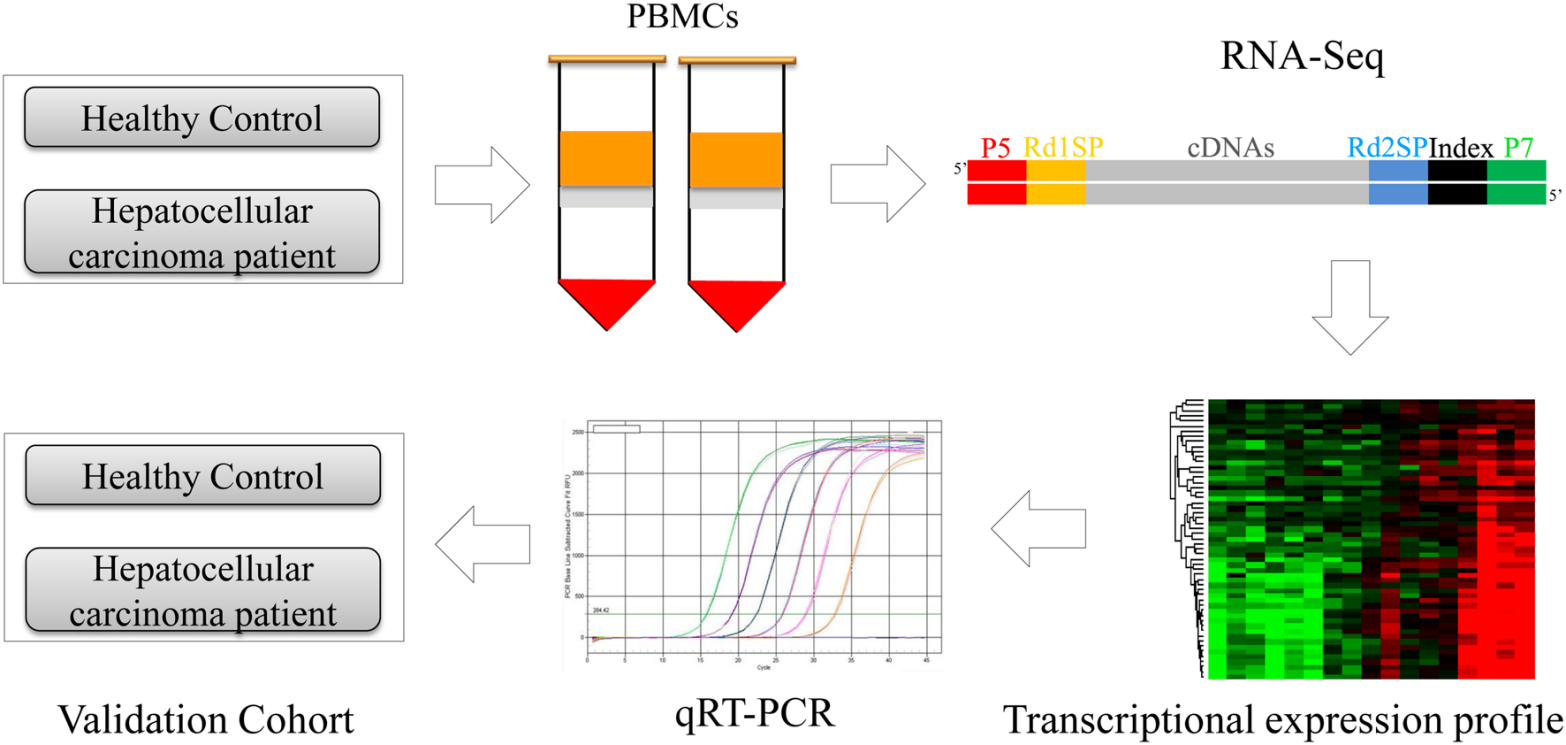
The experiment scheme of the study. It was created using Microsoft office-PowerPoint version 2010, https://www.microsoft.com/zh-cn/microsoft-365/microsoft-office.

Here, we investigated peripheral blood mononuclear cell (PBMC) transcriptomes in HCC and evaluated the diagnostic value of PBMC transcripts. PBMCs, a cell type simple and non-invasive to collect, were isolated from patients with HCC and healthy controls in the present study^13^. Using simple elements for the platform will improve the standard of the solution and yield more reliable results. In addition, RNA-seq was used for the initial screening of dysregulated mRNAs^14^. Then, we performed PCA (principal component analysis) based on the similarities of gene expression and reduced the heterogeneity between tumors by minimizing the size of the effector. Eventually, these methods allowed the path or processes involved in the disorder to be confirmed as a new potential transcript by qRT-PCR. Study design is depicted in Fig. 1. This project will enable us to understand the progression of tumors and lay the foundation for clinical diagnosis in the long run.

Transcriptomes of PBMCs from HCC and control patients were profiled by RNA-seq and then analyzed using bioinformatics methods. Subsequently, several genes were validated by qRT-PCR in an additional validation cohort comprising 33 HCC patients and 32 healthy controls.

## Material and methods

### Isolation of PBMCs and RNA extraction

**Figure 2:**
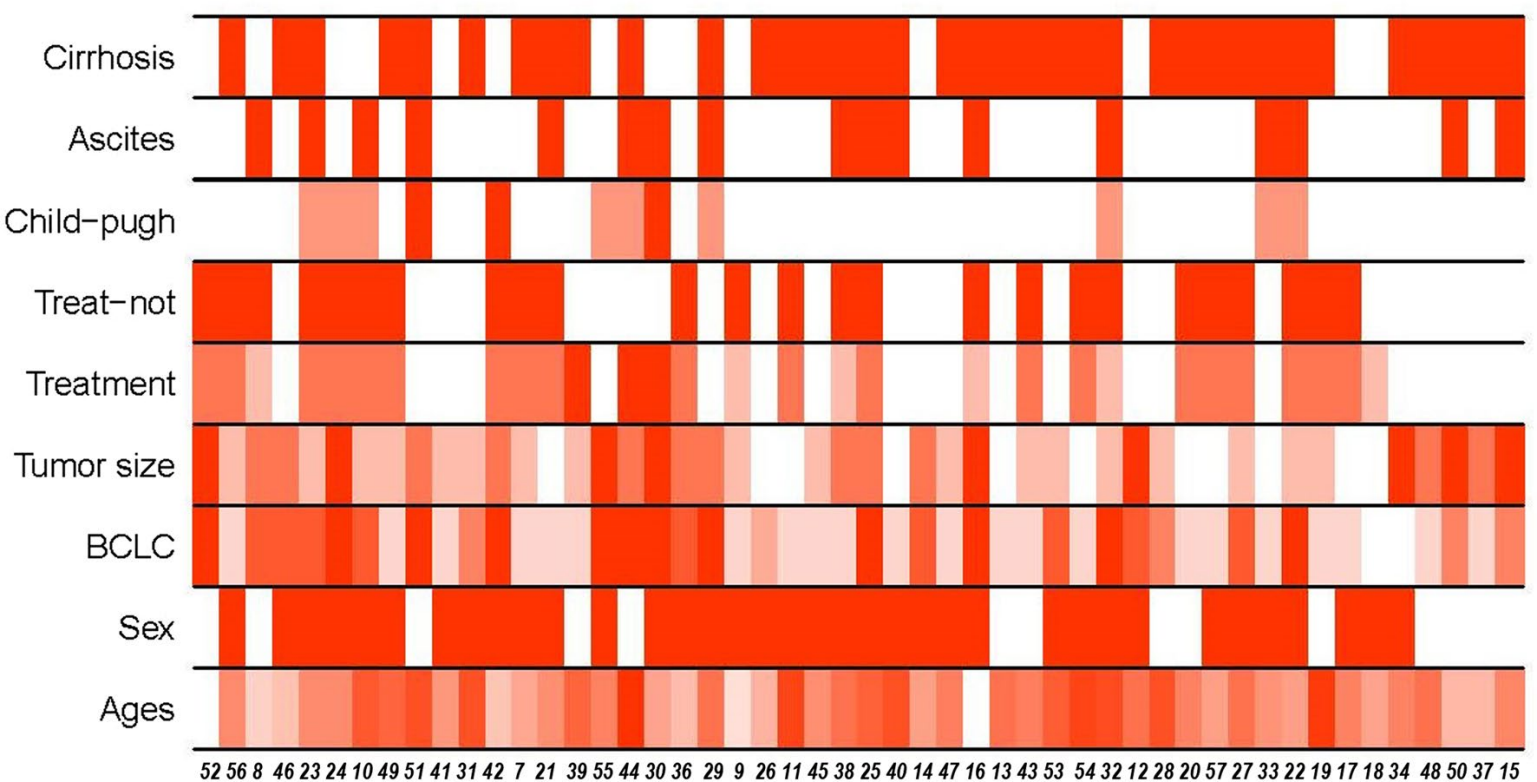
Clinical characteristics of HCC samples, it was created using R version 4.0.2, https://www.r-project.org/. For PBMCs was collected from patients with HCC (17 samples for sequencing and 33 samples for validation). Shown, in descending order, are whether cirrhosis, whether ascites, classification of Child–Pugh, whether treatment or not, treatment method, tumor size, BCLC classification, sex and ages of those patients at time of sampling. The graph was created using R version 4.0.2 (https://www.r-project.org/).

We recruited 34 people from whom peripheral blood (approximately 2 ml) was collected (17 HCC patients and 17 healthy controls with similar sex and age distributions as). Additional samples from another 33 patients and 32 healthy controls were also collected for validation experiments by qRT-PCR. Clinical data were obtained only from the HCC patients. The characteristics of the samples are shown in Table S2, and some of them are displayed in Fig. 2 in gradient color mode. For this demonstration, Ficoll-Paque PREMIUM was utilized to separate PBMCs from 2 ml of EDTA blood from the donor according to the user manual. Subsequently, the PBMC specimens were processed with TRIzol reagent (Invitrogen, Carlsbad, CA) according to a previously reported standard procedure^15^. A NanoDrop ND-1000 (ThermoFisher Scientific, Waltham, MA) was used to determine the quality of RNA at 260 nm (A260) and 280 nm (A280) absorption and to evaluate the integrity of RNA by RNA integrity number (RIN; Agilent 2100 RIN Beta Version Software).

We received ethical clearance to perform this study from the Ethical Committee of Shenzhen Traditional Chinese Medicine Hospital. All experiments were conducted in compliance with the principles and regulations laid down by the ethics committee.

### RNA-seq

Thirty-four RNA samples collected were sent for sequencing. Seventeen of these samples were extracted and matched to healthy controls from patients with HCC. Based on previous studies, the sequencing library was prepared after the removal of rRNA according to the Illumina TruSeq RNA sample preparation guide (Illumina, San Diego, California, USA). The index adaptor was ligated once the double-stranded cDNA had been synthesized. After size selection using Agencourt AMPure XP (Beckman), Qubit 2.0 Fluorometer with Qubit dsDNA HS Analysis Kit (Invitrogen, Eugene, OR, USA) and Agilent Bioanalyzer Quantitative and qualitative library (Agilent Technologies, Santa Clara, CA, USA), samples were submitted to Illumina HiSeq X-10 (Illumina, San Diego, CA, USA) for pair-end sequencing of 2 × 150 bp.

### Bioinformatic analysis

Raw reads were filtered by SOAPnuke (version 1.0.1) and then mapped to the human (hg19) genomes supplied by Illumina iGenomes (Download source: cufflinks.cbcb.umd.edu/igenomes.html) using Tophat2 (version 2.0.7) and the Bowtie2 tool (version 2.1.0) using the default settings. Alignment and differential gene expression analysis were performed using Cufflinks (version 2.0.2)^16^. DEGs demonstrated significant differences if P was less than 0.05 and the fold change was greater than twofold when the results were analyzed.

We utilized the PANTHER (protein annotation through evolutionary relationship) classification system for GO assessment in the functional analysis section (http://www.pantherdb.org/)^17^. Using the Mann–Whitney test, we performed a statistically significant overrepresentation test, which can be used to evaluate whether any ontology class or path has a nonrandomly distributed significance relative to the overall set of attributes. Dysregulated pathway-related HCC-specific genes were analyzed as described in a recent report article by Buzdin A et al.^18^, the activation or inhibition of differentially regulated pathway were denoted by using the pathway activation levels (PALs). Pathway enrich score was calculated with GSVA and differential genes were analyzed by R package limma.

The coexpression modules for DEGs in liver cancer were investigated using Weighted Gene Correlation Network analysis (WGCNA) to examine the link between various modules and clinical properties, such as age, sex, tumor size, and classification. Correlation results are as the main basis for the genes selected for qPCR verification. Then, the protein–protein interaction (PPI) framework of DEGs was visualized using Cytoscape software (version 3.7.2) to gain insights into the involvement of DEGs.

Furthermore, we performed immunoinfiltration analysis using CIBERSORT (https://cibersort.stanford.edu/), and the difference in immune cell populations between HCC and controls, as well as different classifications, was also examined.

### qRT-PCR

For qRT-PCR verification, 65 additional RNA samples were submitted, including 33 HCC patients and 32 healthy controls. Five hundred nanograms of total RNA was submitted for reverse transcription using PrimeScript RT Master Mix (TaKaRa Bio, Inc.), incubation at 37 °C for 15 min and then 85 °C for 5 s to terminate the reaction, with a final volume of 20 μL. The qPCR cycle was conducted using SYBR Premix Ex Taq II (Perfect Real Time; TaKaRa, Bio, Inc.) on an Applied Biosystems 7500 real‑time PCR machine (Life Technologies) using 2 μL of the cDNA obtained in the RT reaction. As shown in Table 1, Sangon Biotech, Inc. synthesized the primers. The PCR took 2 min at 95 °C for predenaturation, then 5 s at 95 °C and 40 s at 60 °C for forty cycles. Three replications were performed for every reaction, with an average Ct value calculated for each triplicate. The ΔCt target cDNA was the difference between the Ct target gene and the Ct reference gene (GAPDH). To assess fold changes in gene expression, the ΔΔCt value between ΔCt of HCC and ΔCt of NC (control sample) was adopted (ΔΔCt = ΔCt HCC − ΔCt NC; ΔCt = Ct target − Ct reference). The quality of the amplified product was determined using a 2% agarose gel and a dissociation curve.

**Table 1:** DNA sequences of the primers used in this study.

Name	DNA sequences of primers
RPL7p24	Forward: CAAGGCTTCGATTAACATGCTGA Reverse: GCCATAACCACGCTTGTAGATT
CALM1	Forward: TTGACTTCCCCGAATTTTTGACT Reverse: GGAATGCCTCACGGATTTCTT
HSPA8P4	Forward: ATGCCAAACGTCTGATTGGAC Reverse: AGCATCATTCACCACCATAAAGG
SLC26A8	Forward: CATGGCACAGGTTCCTACGAT Reverse: GGCCAACACTTATACCAGCAAG
SLC4A1	Forward: CCTATACGCTTCCTCTTTGTGTT Reverse: CCATGTAGGCATCTATGCGGA
SELENBP1	Forward: ACCCAGGGAAGAGATCGTCTA Reverse: ACTTGGGGTCAACATCCACAG
GAPDH	Forward: ACAACTTTGGTATCGTGGAAGG Reverse: GCCATCACGCCACAGTTTC

### Comparison with tumor sequencing data

To confirm the DEGs obtained from PBMCs of HCC patients, we obtained partial sequencing data of HCC tumors from The Cancer Genome Atlas (TCGA) database. Differential gene expression was performed, and expression levels of the six selected genes were also analyzed. The human molecular pathway was also calculated by using PALs as previously described.

### Statistical analysis

All data analyses were performed using R (version 4.0.1) and SPSS (version 22.0). To evaluate the similarity of the samples, we used PCA and Pearson correlation analysis. To evaluate the correlations, Spearman correlation analysis was conducted. A t-test was utilized for determining significant differences. Two-sided inspections were performed in all mathematical inspections. If the p-value was less than 0.05, it was considered statistically significant. Additionally, receiver operating characteristic (ROC) curve analysis was performed to evaluate the diagnostic accuracy of the different genes analyzed.

### Ethics approval

All patients participating in the study provided their informed understanding in writing. Among them, patients under 18 that informed consent were obtained from the parents. Ethics approval was obtained from the Ethics Committee of Shenzhen Traditional Chinese Medicine Hospital. All tests were conducted according to the pertinent guidelines and regulations implemented by this Ethics Committee.

### Consent to participate

All volunteers signed an informed consent to participate in the experimental project.

## Results

### Baseline characteristics and RNA-seq information of samples

RNA-Seq was used to profile gene expression in PBMCs from 17 hepatocellular patients and 17 age-appropriate healthy individuals (as a control group). The raw reads of RNA-seq from 58,012,158 to 83,083,036 are in line with the human reference hg19, which represented readings mapped to exons from 22,894,689 to 42,821,652 (37.420% -57.238%). The read mapping statistics results are shown in Table S3, and the sequencing quality control and alignment regions of the sequencing samples are also presented in the same table. The baseline features of patients with HCC are shown in Table S2. These include infection status, treatment method, tumor sizes, clinical stages, classification, hepatic function index and hematological index of most patients. Among them, age, sex, BCLC, classification information and tumor size of all samples are displayed in gradient color in Fig. 2. The differences in each characteristic can be clearly seen in the figure.

### Characterizing the gene expression profiles of HCC

**Figure 3:**
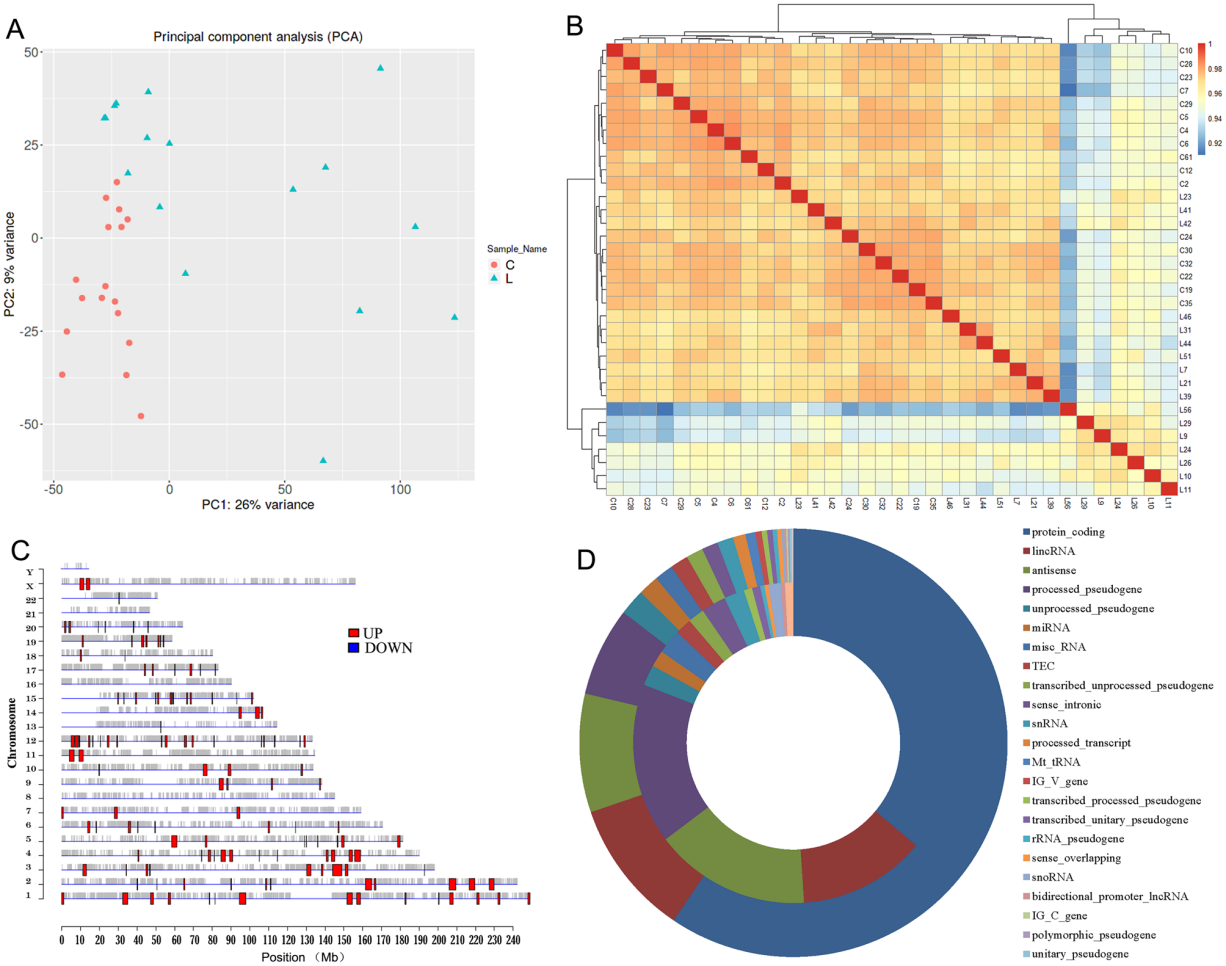
The PCA, heatmap and distribution was created using iDEP version 0.92, http://bioinformatics.sdstate.edu/idep/ and the pie graph was created using Microsoft office-Excel version 2010, https://www.microsoft.com/zh-cn/microsoft-365/microsoft-office. (**A**) Load plot of PCA; (**B**) The pearson’s correlation coefficient of significantly dysregulated mRNA expression the heatmap of inter-sample correlation; (**C**) Distribution of the different expressed genes located in human chromosomes; (**D**) Types of these differential expressed genes sequenced, the outside ring indicated the upregulated genes and the inner ring indicated downregulated genes.

PCA and Pearson’s correlation were used to assess the similarity of the gene expression levels of PBMCs from different HCC patients. PCA is a linear projection methodology that enables researchers to visualize large data in a smaller space. The findings suggested that the first main component (PC1) accounted for 26% of the overall variance of the data, and the second main component (PC2) accounted for 9%. As shown in Fig. 3A, in PC1, the control samples and the HCC samples were gathered. Each had similar effects, indicating their similarity, while PC1 showed the basic features of the expression pattern of HCC. In addition, it has been shown that there are certain distinctions in gene expression profiles between the two groups of PC2 with respect to control samples and cancer samples.

The heatmap of intersample correlation is shown in Fig. 3B. There were obvious differences between the two groups, with no differences found in control samples and only small differences in the HCC group. In addition, DEGs were categorized depending on multiple chromosome positions (Fig. 3C). All chromosomes might show expression of related genes, while almost no DEGs were found on chromosomes 8, 16, 21 or the Y chromosome. Furthermore, Fig. 3D shows the types of these DEGs, which mostly come from protein coding regions, followed by lincRNA, antisense and proceeded by pseudogenes. It is worth noting that few DEGs were from miRNAs, MT_tRNAs or snoRNAs. The molecules identified herein are indicated to have potential roles in HCC incidence and growth.

### DEGs in HCC

**Figure 4:**
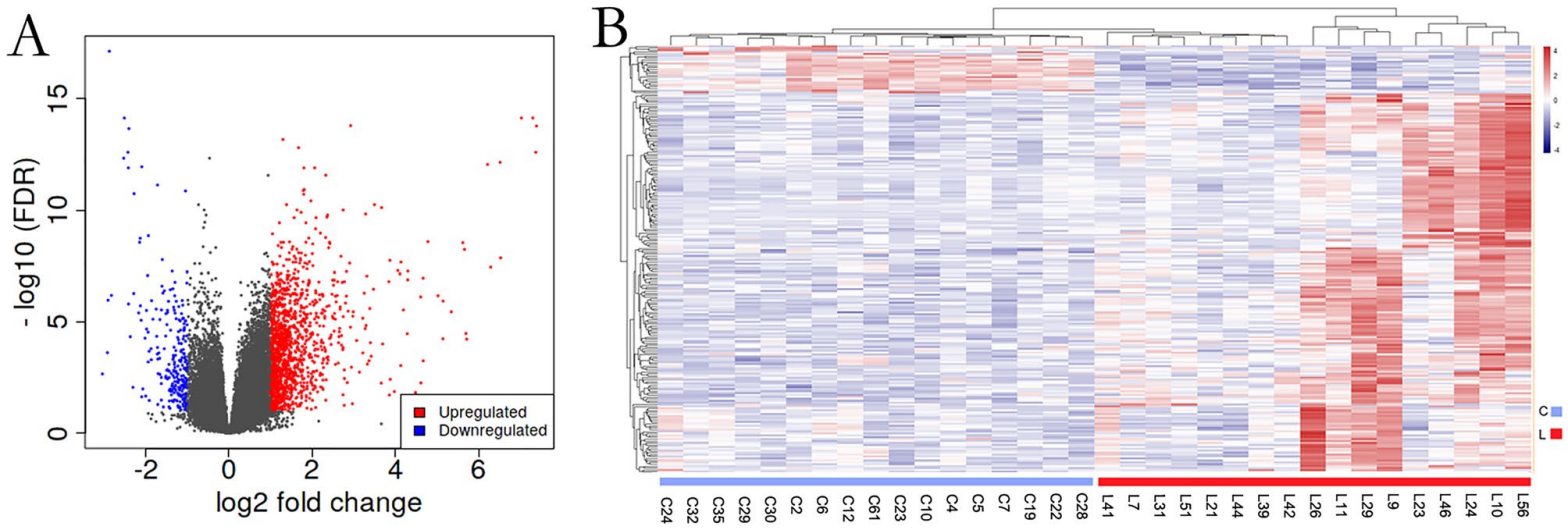
The discrepancy between HCC and healthy controls in terms of gene expression profile. It was created using iDEP version 0.92, http://bioinformatics.sdstate.edu/idep/. (**A**) The plot and the dispersion of the volcano that shows the mRNA differential expression between HCC and NC. The red (up) and blue (down) sites reflect the differentially expressed RNAs with fold threshold ≥ 2.0 and p-Value < 0.05; (**B**) DEGs hierarchical evaluation for clustering in HCC PBMCs.

RNA-seq data identified a total of 60,006 genes in the 34 PBMC samples. Genes that were not expressed in any samples or that were expressed at extremely low levels were removed for subsequent analysis, and only genes with more than 0.5 counts per million in at least one sample were reserved. After filtering, 30,474 genes were retained and are shown in Table S4. Based on the volcano plot, 1,578 genes were dysregulated by a fold change greater than or equal to 2.0 between the HCC and control groups and with a p-value of less than 0.05 (Fig. 4A). Moreover, most DEGs are genes that encode proteins. In HCC patients, 1,334 genes were upregulated and 244 genes were downregulated. The top 25 dysregulated expressed genes are shown in supplemental Table S1. Hierarchical clustering of these dysregulated genes showed that gene expression profiles were distinguishable between the hepatic carcinoma and control groups (Fig. 4B). These results indicate that PBMC RNA from HCC patients is distinctive from that of healthy controls.

### GO and KEGG pathway analysis of DEGs

**Figure 5:**
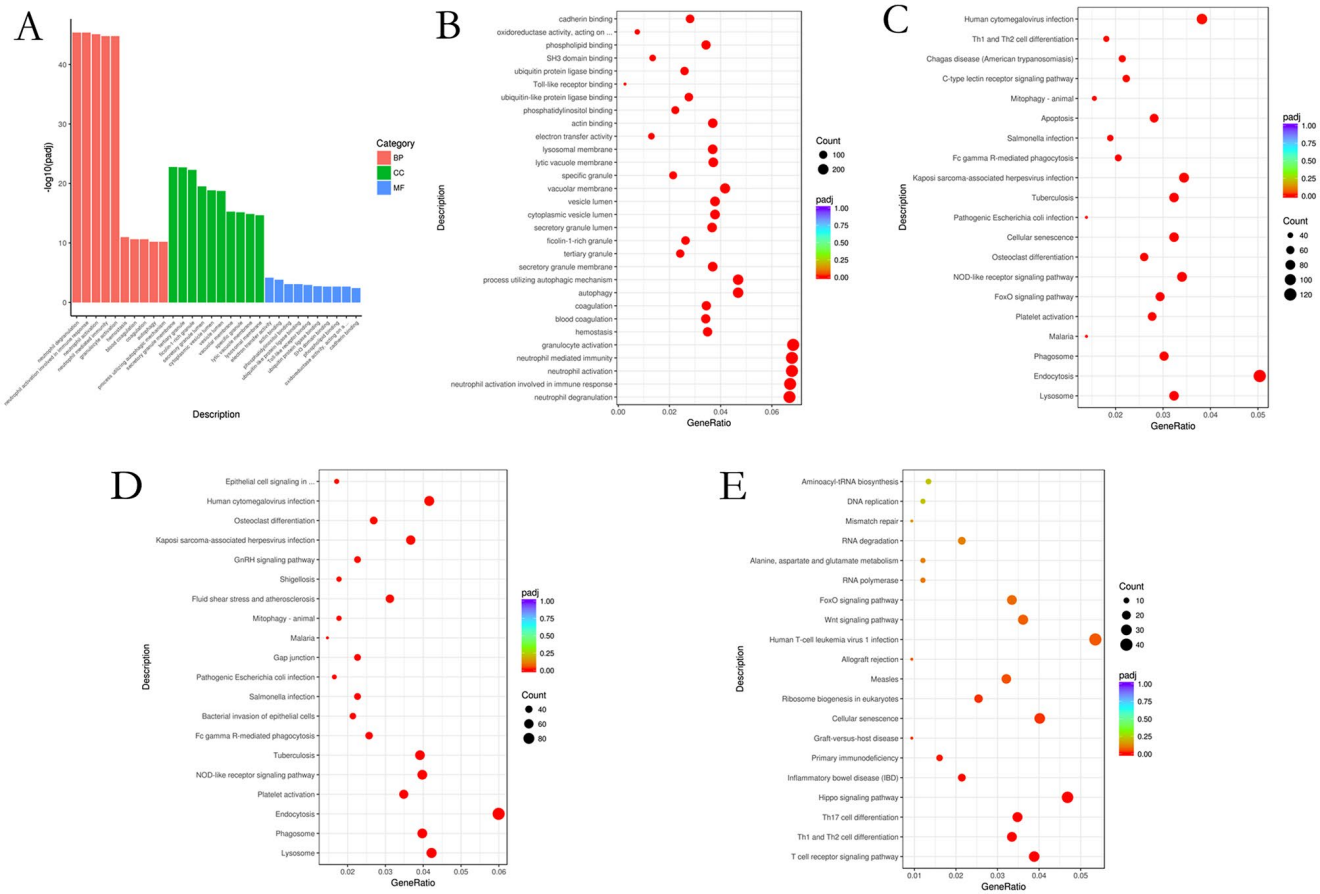
DEGs research for HCC PBMC in terms of GO and KEGG. It was created using R version 4.0.2, https://www.r-project.org/. (**A**) Results of DEGs for GO enrichment research. The biological, cellular, molecular and all-GO-terms in a figure are from left to right with the p-value below 0.05; (**B**) GO term enrichment; (**C**) The top 20 KEGG pathways analysis result of DEGs, the KEGG term with a p-value below 0.01; (**D**) The KEGG pathways analysis result of upregulated expressed genes; (**E**) The KEGG pathways analysis result of downregulated expressed genes.

The identified DEGs are indicated to have possible roles in HCC incidence and progression. As shown in Fig. 5A,B, we discovered that in biological processes, important terms in GO were neutrophil degradation, activation of neutrophils, neutrophil-mediated immunity, neutrophil activation involved in immune response, granulocyte activation, T cell activation, hemostasis, blood coagulation, coagulation, leukocyte differentiation, and platelet degranulation. The significant enriched GO terms in cell component included secretory granule lumen, specific granule, cytoplasmic vesicle lumen, vesicle lumen, tertiary granule, secretory granule membrane, primary lysosome, azurophil granule, azurophil granule lumen. Included in molecular function was nonmembrane spanning protein tyrosine kinase activity, actin adhesion, actin filament binding, SH2 domain binding, phosphatidylinositol binding, Toll-like receptor binding, phospholipid binding, GTPase activator activity, and GTPase regulator activity. Among them, the GO terms of upregulated genes were significantly enriched, primarily related to immune reaction, secretive granule lumen, Toll-like receptor binding and body fluid level regulation, which have been confirmed to be cancer-related. The downregulated gene function enrichment was involved in ribonucleoprotein complex biogenesis, T cell differentiation, T cell receptor complex, and acetyltransferase activity, which are also closely related to hepatic carcinoma.

Based on KEGG annotations, the pathway study revealed the top twenty signal pathways (Fig. 5C), including Lysosome, Endocytosis, Phagosome, Malaria, Platelet activation, FoxO signaling pathway, NOD-like receptor signaling pathway, T cell receptor signaling pathway, Hepatitis B, Apoptosis and so on, which are closely associated with hepatic carcinoma. Figure 5D,E show the upregulated and downregulated pathways, respectively^19^.

To further investigate which regulatory pathways are activated or inhibited, we performed molecular pathway analysis by calculating direct pathway activation levels (PAL) and compared the identified statistically significantly dysregulated pathways. The dysregulated pathways and pathway enrich score from Biocarta, Reactome, KEGG, Qiagen Pathway Central, NCI, and HumanCYC databases are shown in Table S5.

### Coexpression modules analysis of HCC

**Figure 6:**
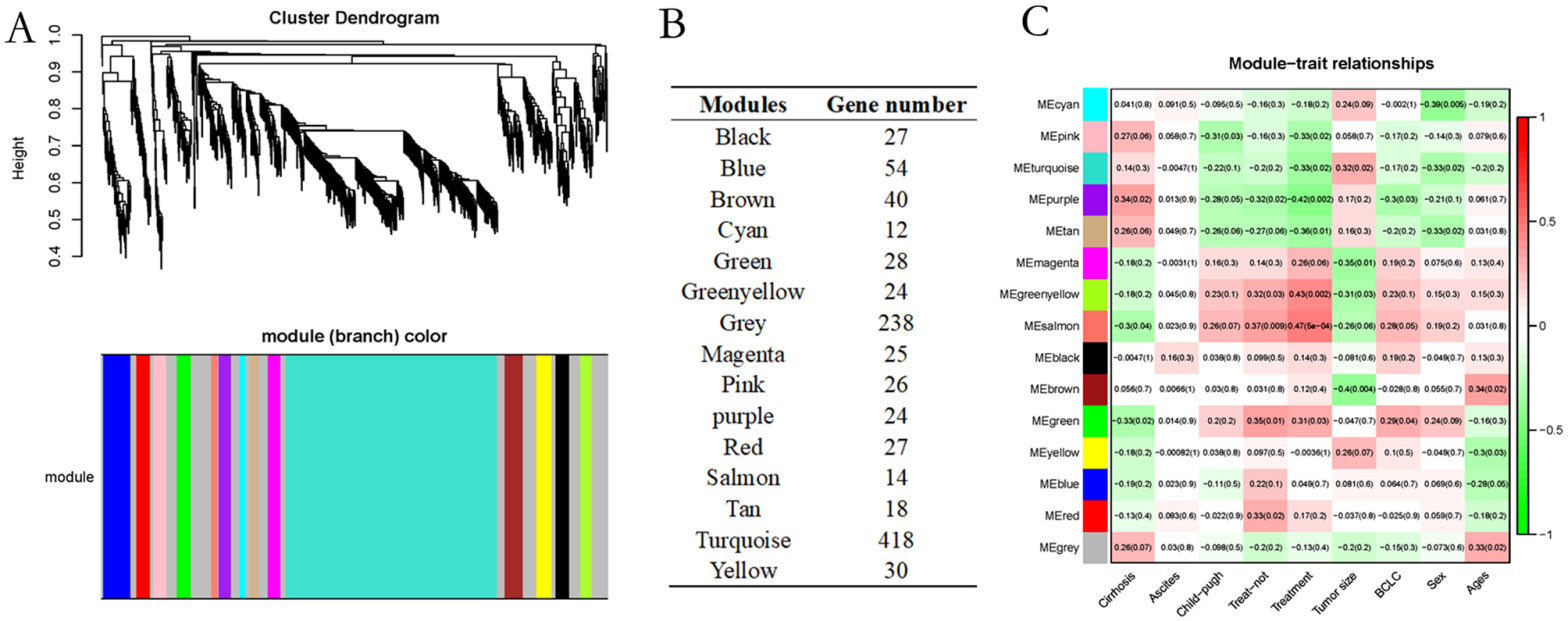
Co-expression modules analysis with WGCNA. It was created using R version 4.0.2, https://www.r-project.org/. (**A**) HCC gene co-expression DEG modules. There are various colors under the dendrogram in the identified modules; (**B**) In the fourteen modules the number of genes appeared on the list; (**C**) Association of module functions. Each row matches a module gene, and it is a function of each column. The respective correlation and p-value are found in any cell (in square brackets). Panel color coding by color legend correlation.

The information contained in PBMC RNA is still complex. For the purpose of more effectively linking information with HCC, WGCNA used DEGs derived from the 17 patients with HCC to formulate a coexpression module. We defined the number of genes in each module at least 10, and the depth of the cutting was 0.8. Finally, 14 gene modules in HCC were defined and are shown in multiple colors (Fig. 6A), and genes not allocated to any of the modules were returned to the gray module. Figure 6B demonstrates the various DEG numbers in the 14 gene modules. Clinical traits, including the age of the patient, sex, tumor size and BCLC classification, were collected, and the correlations between the coexpression module and clinical traits were determined. As shown in Fig. 6C, we found that there were no coexpression modules that had a significant correlation with ascites, suggesting that variously expressed genes in liver cancer have nothing to do with the influence of ascites. From the figure, we can see that these clinical traits had little influence on the HCC DEGs. However, we found that the gene module with salmon was related to several clinical features, such as whether the patient was treated when sampling, the treatment method, whether cirrhosis was present and BCLC classification. Green module gene sets were correlated with cirrhosis, treatment, treatment method and BCLC classification and are worth further analysis.

**Figure 7:**
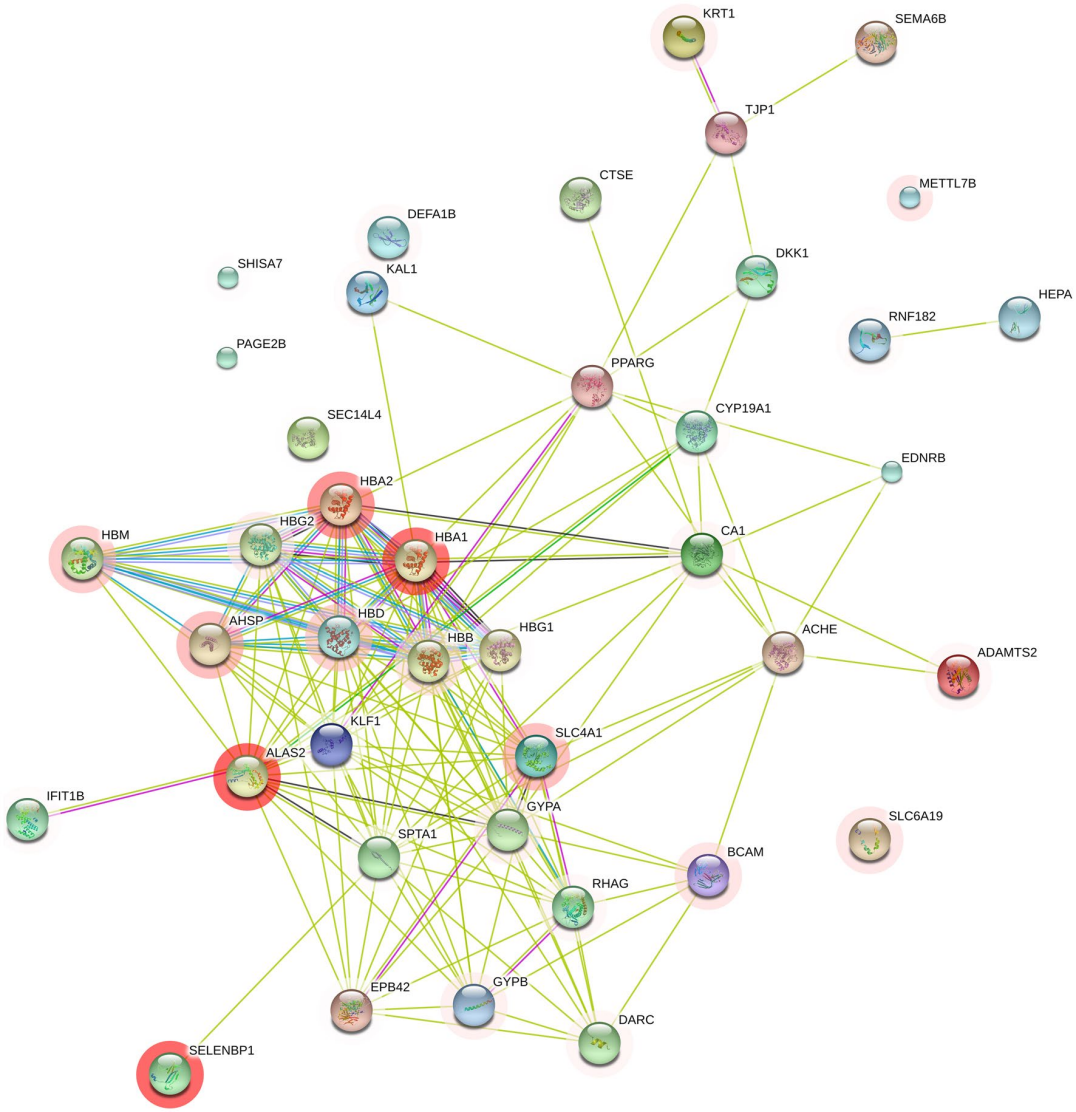
PPI network of DEGs in HCC. It was created using iDEP version 0.92, http://bioinformatics.sdstate.edu/idep/. The ball surrounded by shadows denoted upregulated genes and others showed downregulated genes.

With WGCNA, we found that genes in the blue, turquoise and brown modules occupied the dominance of all the DEG sets, indicating that these genes, such as SELENBP1, SLC4A1, HSPA8P4, CALM1 and CAPN2, CXCR1, and CXCR2, played a marked role in the generation, development and molecular regulation of HCC. It is worth noting that we found that analysis of the module-trait showed that the salmon module had a correlation with several clinical features, including 13 genes contained in this module: AHNAK, CALM1, CAPN2, EEF1A1, HNRNPA1, PPIA, 3 genes from the RPS family (RPS13, RPS14, RPS26) and 4 genes related to RPL, including RPL15, RPL29, RPL7p24 and RPLP0. The results of genetic functional enrichment research indicate that most of the genes are associated with neutrophil-mediated immunity, leukocyte-mediated immunity, leukocyte activation, immune system processes and cell migration, adhesion and motility. The protein–protein interaction (PPI) system of DEGs was formulated from the top 40 DEGs (ranked by fold change). Figure 7 shows that most of the genes interact with others, and only 5 genes have no interactions with others.

### Validation with quantitative RT-PCR

To verify the sequence performance, qRT-PCR was adopted to verify the expression of DEGs in PBMCs of additional HCC patients (n = 33) and a healthy control group (n = 32). Six genes were selected for detection in the validation cohort. WGCNA results are the main basis for the genes selected for qPCR verification. The size of the tumor and clinical stages are the prior factors in the selection. As seen in Fig. 6C, the significantly correlated gene clusters with tumor sizes are labeled in brown, green, yellow, magenta and turquoise. Among these gene clusters, we could find only 4 genes (SELENBP1, SLC4A1, HSPA8P4, SLC26A8) listed in top 25 differential genes (Table S1), which are all in turquoise gene clusters and these 4 genes are selected. We found that other clinical trait such as gender, age, treatment, cirrhosis are significantly correlated with gene clusters labeled in salmon, black, green and purple which might also closely related to HCC. However, there are no genes found in the top 25 differential genes from these cluster. We randomly chose two of them for subsequent verification, one of which is found in both salmon and turquoise (CALM1), the other (RPL7p24) is found in both salmon and green. In addition, several genes, such as CALM1, have been previously reported to be closely related to HCC^20^.

**Figure 8:**
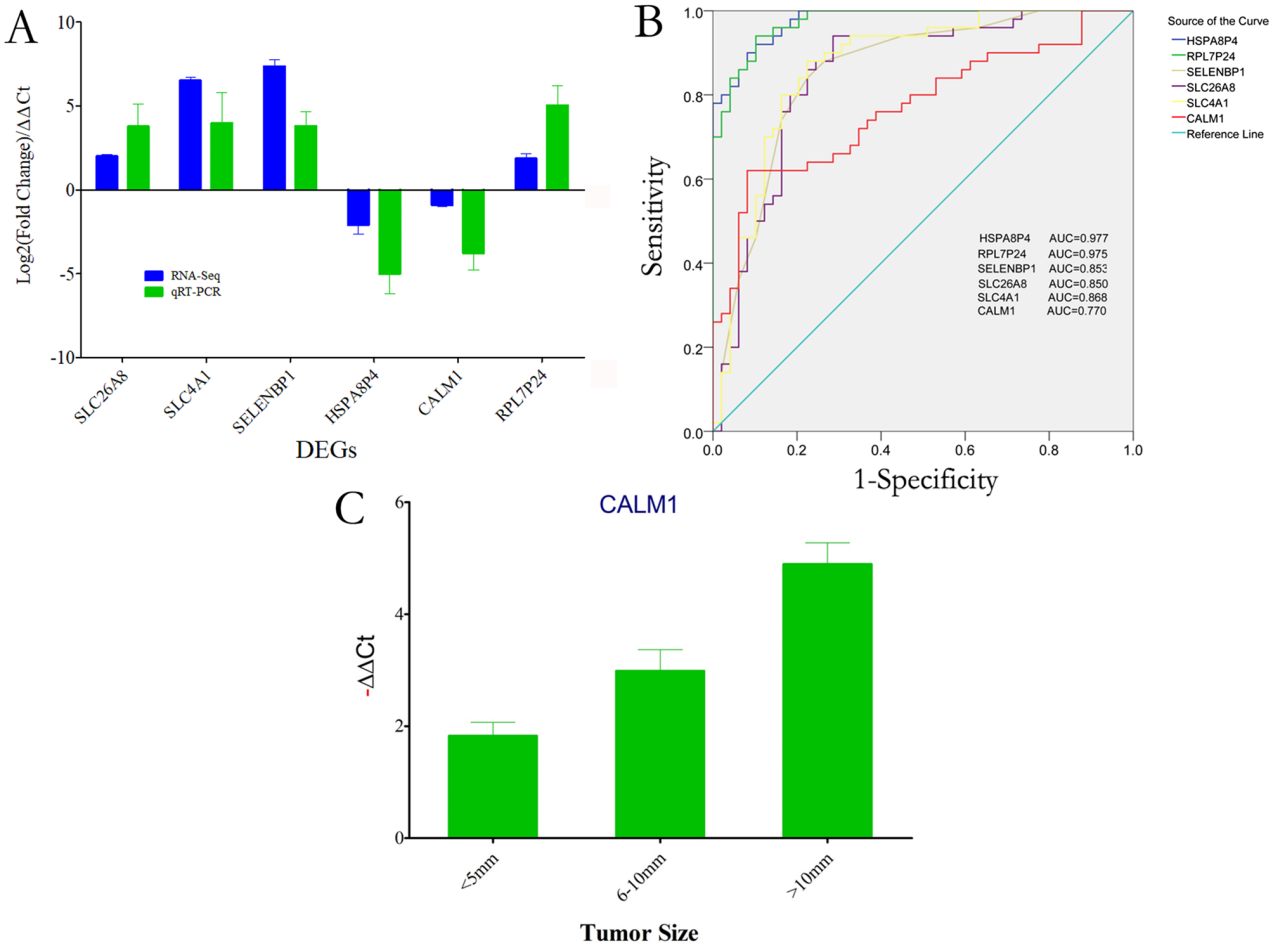
The validation of the PBMC RNA by qRT-PCR. The histogram was created using GraphPad Prism 5, version 5.01, https://www.graphpad.com/scientific-software/prism/ and the ROC curve was created using SPSS version 22.0, https://www.ibm.com/cn-zh/analytics/spss-statistics-software. (**A**) In 33 HCC patients with 32 normal controls, expressions of selected dysregulated genes were assessed employing qRT-PCR in specimens from HCC samples. (**B**) ROC curve analysis of SELENBP1, SLC4A1, SLC26A8, HSPA8P4, CALM1, and RPL7p24, AUC values are given on the graphs. (**C**) Relationship of CALM1 gene expression and tumor sizes, the gene decreased gradually when tumor enlarged.

The sequencing results showed that all 6 genes, except CALM1 (P = 0.063), were significantly differentially expressed in PBMCs of patients with HCC when compared to controls, as shown in Fig. 8A. qRT-PCR results revealed similar changes, and significant differences were found between the HCC group and the normal control group in all 6 genes.

To investigate the diagnostic value of these DEGs for in HCC patients, we performed ROC curve assessment. As shown in Fig. 8B, the AUC was 0.977 (95% CI 0.956–0.998 p < 0.001) for HSPA8P4, 0.975 (95% CI 0.952–0.998 p < 0.001) for RPL7p24, 0.853 (95% CI 0.776–0.931 p < 0.001) for SELENBP1, 0.850 (95% CI 0.770–0.930 p < 0.001) for SLC26A8, 0.868 (95% CI 0.794–0.942 p < 0.001) for SLC4A1 and 0.770 (95% CI 0.676–0.863, p < 0.01) for CALM1. The findings demonstrate that these genes have prospective diagnostic value in patients with liver cancer.

It should be noted that CALM1 expression detected by qRT-PCR was significantly decreased when compared to controls, although no significant differences were found in the sequencing results. More interestingly, expression levels gradually decreased with increasing tumor size (*p-*value < 0.05) (Fig. 8C). These findings suggest that CALM1 might be closely related to HCC tumor development.

### Comparison to sequencing results from tumor samples

**Figure 9:**
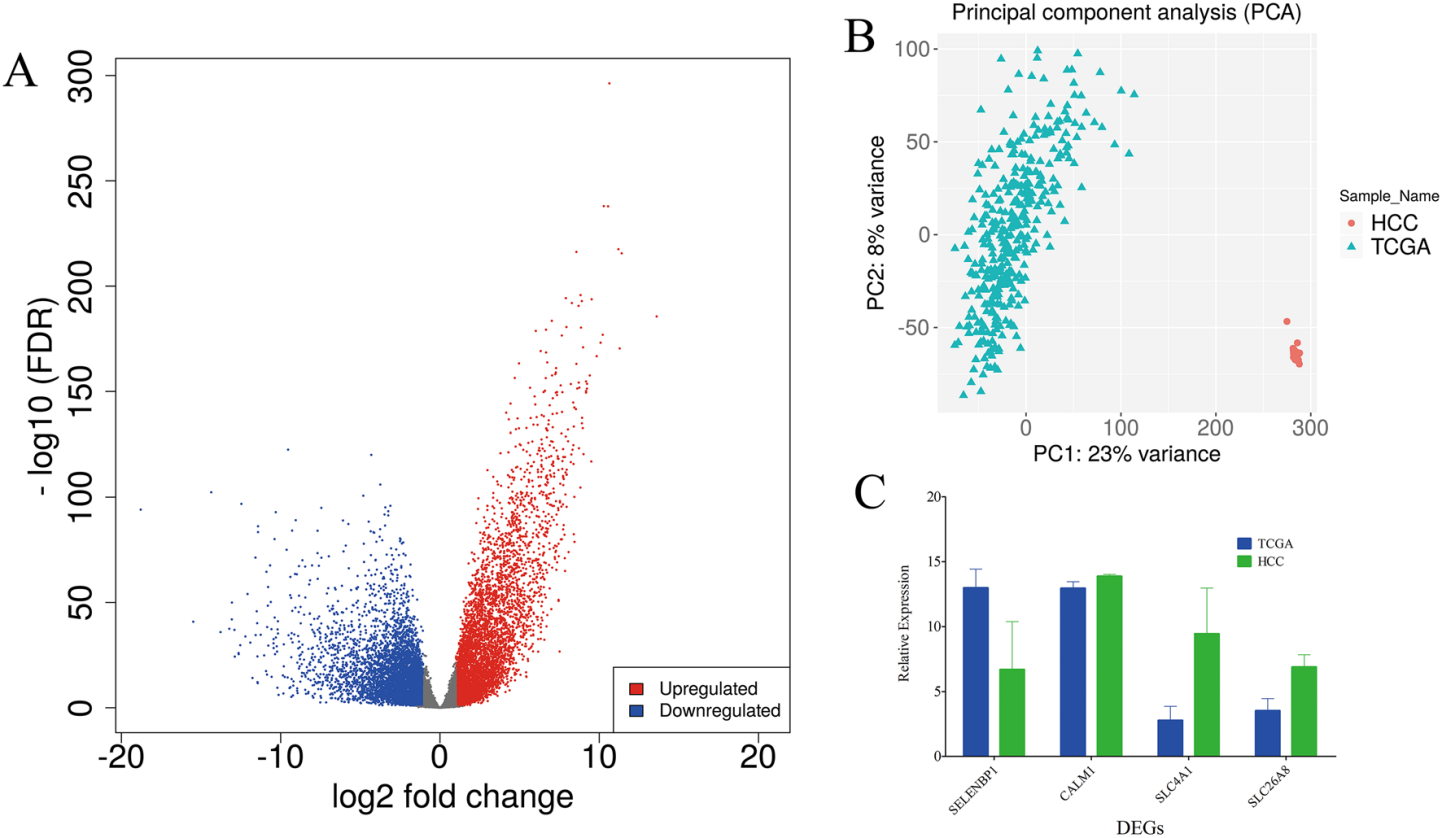
PBMCs and tumor DEGs comparison. The volcano plot and PCA was created using iDEP version 0.92, http://bioinformatics.sdstate.edu/idep/ and the histogram was created using GraphPad Prism version 5.01, https://www.graphpad.com/scientific-software/prism/. (**A**) Volcano plot display of DEGs between PBMCs and tumors; (**B**) PCA of sequencing result of PBMCs and tumors; (**C**) The expression level comparison of genes selected for validation, four genes found co-expression in PBMCs and tumors, SELENBP1, SLC4A1, SLC26A8 except CALM1 showed significantly differences.

To comprehensively investigate DEGs in HCC, comparisons between PBMCs and tissues were also performed. We downloaded matched sequencing data of HCC tumors from The Cancer Genome Atlas (TCGA) database. A total of 14,711 genes were coexpressed in these tumors and the PBMCs used in this study. Volcano plot and PCA results showed that significant DEGs existed between PBMCs and tissues of HCC patients (Fig. 9A,B), and the opposite pattern was observed for gene expression (Figure S1). The DEGs between PBMCs and controls were negligible for the large differences between tumor tissues and PBMCs as shown in Figure S1.

It is worth noting that the six genes selected for validation were also found to be coexpressed in tumor samples, except for RPL7p24 and HSPA8P4. Significant differences were found between PBMCs and tumors in SLC4A1, SLC26A8 and SELENBP1 levels (Fig. 9C). The comparison results of PBMCs and tumors further suggest that these genes could act as potential diagnostic markers. It is inferred that RPL7p24 and HSPA8P4 are expressed in tumors at very low levels or exhibit no expression and could not be sequenced, although the heat shock protein family was reportedly expressed in HCC in a previous study^21,22^.

### Immunoinfiltration analysis

**Figure 10:**
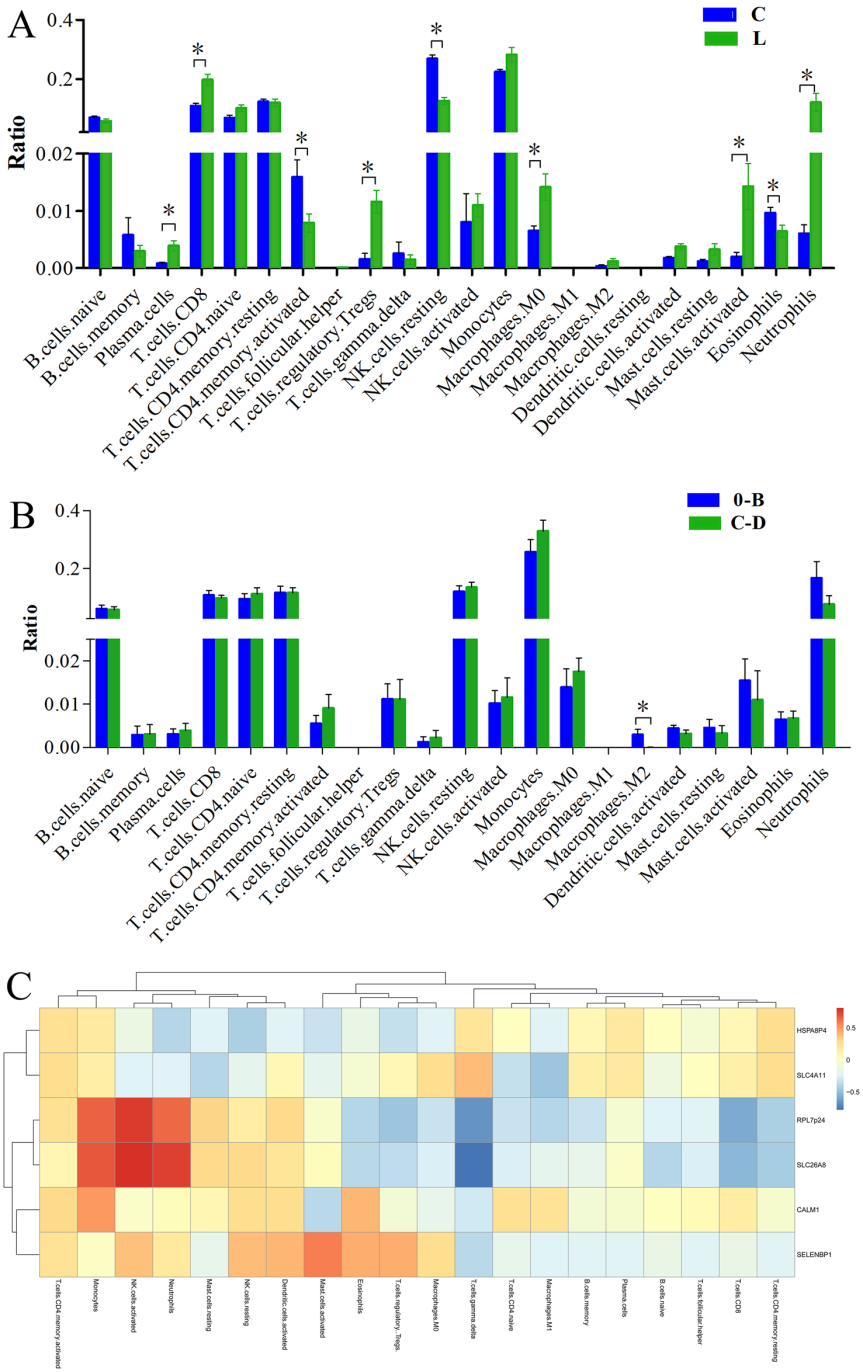
Immunoinfiltration analysis. The histogram was created using GraphPad Prism 5, version 5.01, https://www.graphpad.com/scientific-software/prism/; the heatmap was created using R version 4.0.2, https://www.r-project.org/. (**A**) Immune cell population ratio comparison between HCC patients and controls; (**B**) The cell population differences of different BCLC classification; (**C**) Pearson correlations analysis of the mentioned genes and different immune cells.

To investigate the role of DEGs in PBMCs of HCC, immunoinfiltration analysis was performed. The results showed that most immune cell populations were found in both HCC and control samples. Among them, CD8 T cells, plasma cells, naive CD4 memory cells, resting NK cells and neutrophils exhibited significant differences between HCC patients and controls (Fig. 10A). There was almost no difference among different BCLC stages in these immune cell proportions, except in macrophages (Fig. 10B). Analytic results of the correlation between the DEGs and immune cells showed that RPL7P24 and SLC26A8 were significantly positively correlated with monocytes, NK cells and neutrophils and negatively correlated with CD8 T cells and resting memory CD4 T cells (Fig. 10C).

## Discussion

In this study, PBMCs were separated for RNA-seq to profile differential gene expression in HCC patients. PBMCs primarily contain lymphocytes and monocytes separated from blood samples and are widely used in clinical diagnosis and academic research. It has been confirmed that PBMCs is strongly linked to the occurrence, growth, metastases and prognosis of tumors caused by irregular immune function. The detection of differential gene expression in PBMCs might dictate tumor development. Furthermore, PBMCs are easy to collect and have low complexity, so they would be a suitable choice for studying gene expression and monitoring tumors.

Using RNA-seq, we examined 1,334 upregulated and 244 downregulated genes in PBMCs from liver cancer patients, each of which had a fold change greater than or equal to 2.0 and a p-value below 0.05. The defined DEGs might be generated from tumor cells circulating in the vasculature and therefore circulating throughout the blood^23^.

After GO, KEGG and coexpression analyses, six DEGs were selected for qPCR validation: 4 genes (SELENBP1, SLC4A1, SLC26A8 and HSPA8P4) were selected as the top dysregulated genes and dominated the gene regulation network, and 2 genes (CALM1 and RPL7p24) were selected as WGCNA modules. CALM1 and RPL7p24 were selected from the salmon modules (Fig. 6C), which correlated with treatment method, cirrhosis and BCLC classification. Among them, SELENBP1, SLC4A1, SLC26A8 and RPL7p24 were upregulated, and HSPA8P4 and CALM1 were downregulated according to the sequencing results. All of these genes are involved in regulatory pathways, including the immune response, granulocyte activation, T cell activation, Toll-like receptor binding, and GTPase regulator activity, which have been shown to be closely related to HCC^24–26^.

It is important to find the regulatory pathways that DEGs involved. In this study, we use the latest reported algorithm for human molecular pathways annotation. The pathways of activation and inhibition were obtained by calculating PALs. It can be seen from Table S5 that the pathways such as L-serine degradation are significantly activated. Recently, Yu’s team reported that serine metabolism can regulate the innate immunity of antiviral through inhibit YAP lysosomal degradation^27^. It is suggested that HCC is closely related to immunity. Furthermore, molecular pathways such as Toll-like receptor, transport function of erythrocytes, IL-6, IL-10 and IGF2BP are also significantly activated and list in top when ranked by PALs. While the molecular pathway such as IL-12, basal cell carcinoma, estrogen receptor, Ephrin A, PIK3, CD8, CD4 and IGF1R were significantly inhibited. Most of them are closely related to immune functions might act important roles in the occurrence and development of HCC. The PALs and related molecular pathways were also list in the table when compared with tumor sequencing data obtained from TCGA database. Among them, Mismatch repair, PLK1, tumor suppressor arf inhibits ribosomal biogenesis, viral mRNA translation, phosphorylation was activated and FoxA1 transcription factor, IGF1R, Estrogen receptor, cell adhesion, VEGF and so on was significantly inhibited. It is shown that there are some differences between PBMCs and tumor in the molecular pathways, because the PBMCs showed all the changes in whole body and the tissue results only represent tumor changes. It can be inferred that those shared pathways might promote the tumorigenesis and development.

The expression of all six genes in HCC samples was confirmed by qRT-PCR. We found that no previous studies had shown that expression of SLC4A1 and RPL7p24 was related to HCC, although mutations in these genes have been reported in many diseases^28–30^. Calmodulin-related genes have been implicated in many cancers, and CALML3 was reported to be a potential biomarker for pulmonary metastasis of HCC^20^, while dysregulation of CALM1 in HCC was first observed in our previous study^31^. Increased expression of HSPA8 was reported in HCC and depressive disorder^32^, and previous studies also showed that SLC26A8 (solute carrier family 26 member 8) is related to many cancers, including colorectal cancer, with mutation of SLC26A8 being related to many diseases^33–35^.

Notably, SELENBP1 (selenium binding protein 1) has been reported to be downregulated in colorectal cancer but upregulated in HCC in this study, and this was confirmed by qRT-PCR in the validation cohort^36^. As shown in Fig. 8B, higher specificity and sensitivity were obtained when distinguishing liver cancer from normal samples based on the expression level of SELENBP1.

In the present study, CALM1 was downregulated in HCC patients, while the *p*-value was 0.063, which indicated no significant differences. However, we still selected it for two reasons: one is because it was found in the gene sets from WGCNA modules, and the other is the gradual changes found CALM1 expression in patients with tumor sizes from small to large. As expected, significant differences were obtained from qRT-PCR between liver cancer patients and normal controls. The gradually decreasing expression of CALM1 was also confirmed by RT-PCR, as shown in Fig. 8C.

It cannot be denied that there are still limitations in this study. There were no sequencing results obtained from tissues of HCC patients. Therefore, we downloaded matched data from TCGA database for comparison; results showed almost the opposite expression mode compared to PBMCs. It is inferred that the significant differences might be primarily due to immune-related genes highly expressed in PBMCs but downregulated in tumor tissue samples due to immune infiltration. A recent study showed similar results, in which expression of immune checkpoint genes and their roles in predicting the immunotherapy response^37,38^ and immune-related genes can differentiate tumor cells and immune cells very well.

Additionally, the population of immune cells was also calculated for each sample in the present study. A significant difference was found in the population of immune cells between HCC and control samples. It was further demonstrated that immune cells play an extremely important role in the occurrence of HCC, especially CD8^+^ T cells, which has been confirmed by previous studies with respect to determining prognosis. We also found that several patients died within twelve months and had a higher ratio of CD8^+^ T cells. However, there was no statistical analysis due to the small sample size^39,40^.

On the other hand, the patient cohort in this study was relatively small. There were too many physiological differences among these patients with HCC, although 17 samples were sequenced, and only a few samples could be used for comparison when analyzing the correlations between traits and different gene modules, leading to the current results not being reliable enough. However, potential biomarkers could be screened from the DEGs obtained by PBMC RNA sequencing for HCC.

## Conclusions

Identified DEGs were profiled by RNA-seq from PBMCs of HCC patients in the present study. A total of 1,578 DEGs were found between HCC and healthy controls, including 1,334 upregulated genes and 244 downregulated genes. Functional analysis of gene expression in HCC revealed that the majority of genes in the HCC samples were related to immune responses. Several DEGs selected (SELENBP1, SLC4A1, SLC26A8, HSPA8P4, CALM1 and RPL7p24) were confirmed by qRT-PCR, and to our knowledge, SLC4A1, RPL7p24, CALM1 and SLC26A8 were first found to be related to HCC, suggesting that potential biomarkers could be analyzed for the classification, stages and therapeutic target of HCC in future studies.

## Supplementary Information

Supplementary Information.

Supplementary Figure S1.

Supplementary Table S1.

Supplementary Table S2.

Supplementary Table S3.

Supplementary Table S4.

Supplementary Table S5.

## Data Availability

All test statistic produced or evaluated in the course of this research is disclosed in the paper as well as its supplementary information files. The original sequencing data from this research are available at NCBI project PRJNA739257 (https://dataview.ncbi.nlm.nih.gov/object/PRJNA739257).
