# Supplementary material for: RNA-seq profiling reveals PBMC RNA as a potential biomarker for hepatocellular carcinoma
Source: Sci Rep. 2021 Sep 7;11:17797. doi: 10.1038/s41598-021-96952-x (PMC8423838; doi:10.1038/s41598-021-96952-x)
Supplement: Supplementary file 1 — Supplementary Information. [file 41598_2021_96952_MOESM1_ESM.docx]

**Supplement figure legends**

**Figure.S1** Comparison between PBMCs and tumors, hierarchical clustering, PCA and venn diagram of the different expressed genes. It was created using iDEP version 0.92, <http://bioinformatics.sdstate.edu/idep/>. (A) The hierarchical evaluation for clustering in PBMCs from HCC compared with tumor from HCC. Significant gene expression differences found between tumor samples and PBMCs. (B) PCA of sequencing results of HCC from TCGA database, PBMCs of HCC patient and healthy controls. The expression differences of PBMCs between HCC and controls were masked by the expression of tumor tissues. (C) Venn diagram of DEGs between tumor, PBMCs and PBMC controls.

**Supplement Tables**

**Table S1** The top 25 dysregulated gene list identified from PBMC samples from patient with HCC using RNA-Seq.

**Table S2** All available clinical character information from patients with HCC.

**Table S3** The PBMCs transcriptome sequencing read mapping statistics results.

**Table S4** Expression of matrix of PBMCs from patient with HCC sequenced in the study.

**Table S5** The dysregulated pathways and pathway enrich score from Biocarta, Reactome, KEGG, Qiagen Pathway Central, NCI, and HumanCYC databases.
