# Supplementary figures and images for: RNA-seq profiling reveals PBMC RNA as a potential biomarker for hepatocellular carcinoma
Source: Sci Rep. 2021 Sep 7;11:17797. doi: 10.1038/s41598-021-96952-x (PMC8423838; doi:10.1038/s41598-021-96952-x)

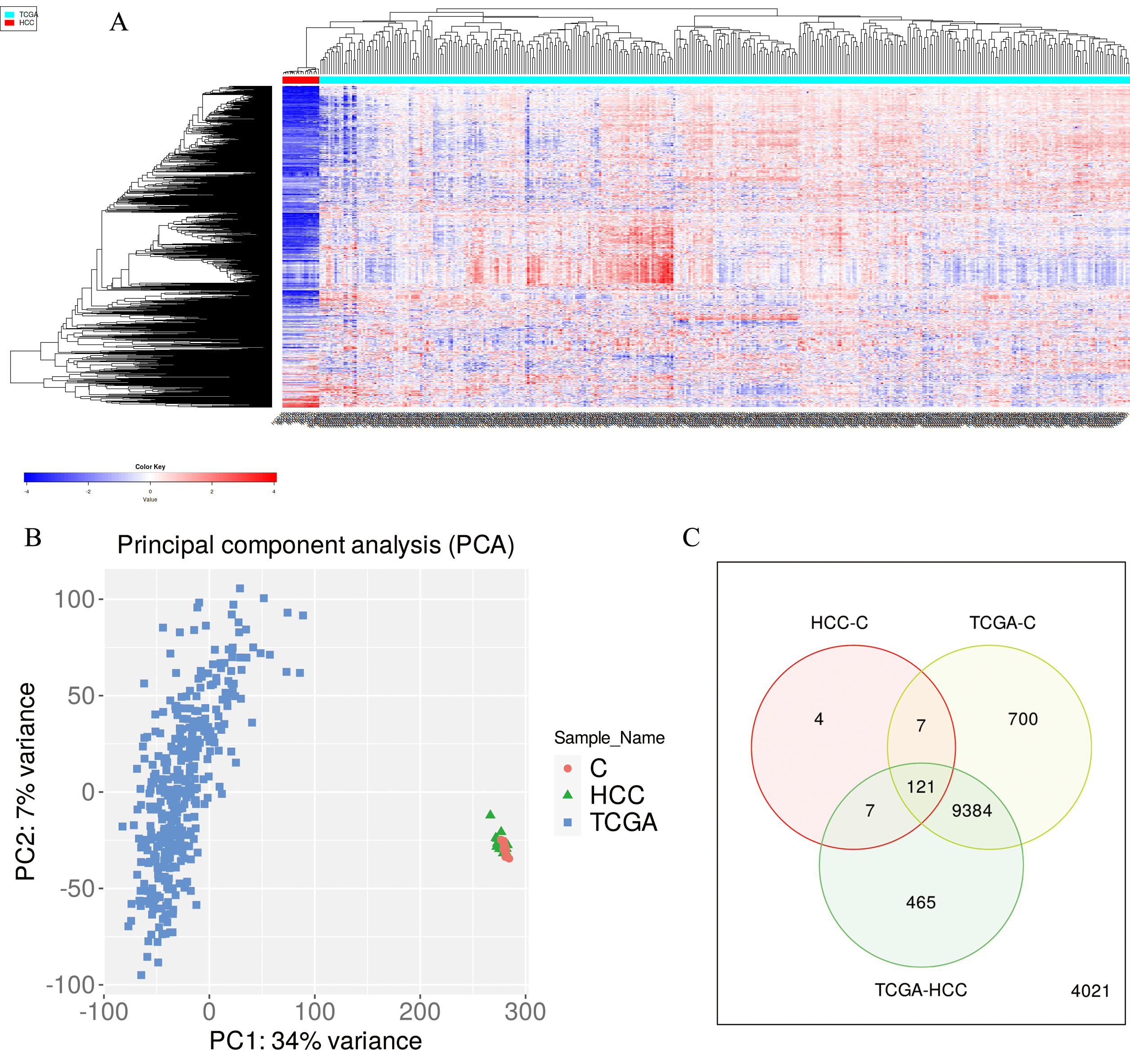

Supplement: Supplementary file 2 — Supplementary Figure S1. [file 41598_2021_96952_MOESM2_ESM.tif]
