# Supplementary material for: RNA-seq profiling reveals PBMC RNA as a potential biomarker for hepatocellular carcinoma
Source: Sci Rep. 2021 Sep 7;11:17797. doi: 10.1038/s41598-021-96952-x (PMC8423838; doi:10.1038/s41598-021-96952-x)
Supplement: Supplementary file 3 — Supplementary Table S1. [file 41598_2021_96952_MOESM3_ESM.docx]

Table S1 The top dysregulated gene list

| Symbol | Log2FoldChange | adj *p* value | Regulation direction |
| --- | --- | --- | --- |
| ALAS2 | 7.389014323 | 1.64E-14 | up |
| SELENBP1 | 7.370383076 | 2.51E-13 | up |
| HBA1 | 7.298641092 | 7.16E-15 | up |
| HBA2 | 7.023682642 | 7.16E-15 | up |
| AHSP | 6.525679283 | 1.40E-08 | up |
| SLC4A1 | 6.512977499 | 7.09E-13 | up |
| HBM | 6.286855013 | 3.62E-08 | up |
| HBD | 6.211237725 | 8.80E-13 | up |
| ABCC13 | 5.710552502 | 5.99E-05 | up |
| BCAM | 5.692377878 | 3.34E-05 | up |
| METTL7B | 5.656415168 | 5.77E-09 | up |
| SLC6A19 | 5.62591098 | 0.046801069 | up |
| HBB | 5.618061912 | 2.88E-09 | up |
| HBG2 | 5.340603255 | 3.55E-06 | up |
| GYPB | 5.142991414 | 1.17E-06 | up |
| SLC26A8 | 2.000982705 | 8.97E-09 | down |
| ITGA9 | 2.000908248 | 3.06E-05 | down |
| TACSTD2 | 2.000126436 | 0.007133033 | down |
| HSPA8P4 | -2.071093741 | 0.010424724 | down |
| C10orf111 | -2.095872502 | 1.12E-12 | down |
| DNAJB5-DT | -2.111450159 | 8.54E-06 | down |
| SLC25A24P1 | -2.308492356 | 0.008892559 | down |
| OSR2 | -2.372874596 | 4.68E-05 | down |
| LINC02399 | -2.529317278 | 4.62E-13 | down |
| HERC2P10 | -2.824212755 | 6.53E-07 | down |
